# Heterologous expression and structure prediction of a xylanase identified from a compost metagenomic library

**DOI:** 10.1007/s00253-024-13169-4

**Published:** 2024-05-10

**Authors:** Joana Sousa, Cátia Santos-Pereira, Joana S. Gomes, Ângela M. A. Costa, Andréia O. Santos, Ricardo Franco-Duarte, João M. M. Linhares, Sérgio F. Sousa, Sara C. Silvério, Lígia R. Rodrigues

**Affiliations:** 1 CEB - Centre of Biological Engineering, Universidade do Minho, Campus de Gualtar, 4710-057 Braga, Portugal; 2 LABBELS - Associate Laboratory, Braga/Guimarães, Portugal; 3 CBMA - Centre of Molecular and Environmental Biology, Department of Biology, University of Minho, Campus de Gualtar, 4710-057 Braga, Portugal; 4 IB-S - Institute of Science and Innovation for Bio-Sustainability, University of Minho, Campus de Gualtar, 4710-057 Braga, Portugal; 5 Physics Center of Minho and Porto Universities (CF-UM-UP), Universidade do Minho, Campus de Gualtar, 4710-057 Braga, Portugal; 6 LAQV/REQUIMTE BioSIM – Department of Biomedicine, Faculty of Medicine, University of Porto, 4200-319 Porto, Portugal

**Keywords:** Composting, Glycosyl hydrolases, Metagenomics, Molecular docking, Xylanases

## Abstract

**Abstract:**

Xylanases are key biocatalysts in the degradation of the β‐1,4‐glycosidic linkages in the xylan backbone of hemicellulose. These enzymes are potentially applied in a wide range of bioprocessing industries under harsh conditions. Metagenomics has emerged as powerful tools for the bioprospection and discovery of interesting bioactive molecules from extreme ecosystems with unique features, such as high temperatures. In this study, an innovative combination of function-driven screening of a compost metagenomic library and automatic extraction of halo areas with in-house MATLAB functions resulted in the identification of a promising clone with xylanase activity (LP4). The LP4 clone proved to be an effective xylanase producer under submerged fermentation conditions. Sequence and phylogenetic analyses revealed that the xylanase, Xyl4, corresponded to an endo-1,4-β-xylanase belonging to glycosyl hydrolase family 10 (GH10). When *xyl4* was expressed in *Escherichia coli* BL21(DE3), the enzyme activity increased about 2-fold compared to the LP4 clone. To get insight on the interaction of the enzyme with the substrate and establish possible strategies to improve its activity, the structure of Xyl4 was predicted, refined, and docked with xylohexaose. Our data unveiled, for the first time, the relevance of the amino acids Glu133 and Glu238 for catalysis, and a close inspection of the catalytic site suggested that the replacement of Phe316 by a bulkier Trp may improve Xyl4 activity. Our current findings contribute to enhancing the catalytic performance of Xyl4 towards industrial applications.

**Key points:**

• *A GH10 endo-1,4-β-xylanase (Xyl4) was isolated from a compost metagenomic library*

• *MATLAB’s in-house functions were developed to identify the xylanase-producing clones*

• *Computational analysis showed that Glu133 and Glu238 are crucial residues for catalysis*

**Graphical abstract:**

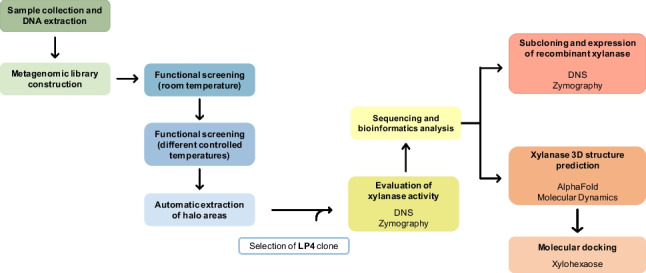

## Introduction

The overexploitation of global non-renewable resources, particularly fossil fuels, has caused negative effects on the natural ecosystems that, consequently, disturb the quality of human health (Mujtaba et al. 2023). The valorization of the lignocellulosic biomass as a sustainable alternative to mitigate environmental and health issues and reduce the dependence on carbon fossil resources has gained the attention of both the scientific community and the industry (Dutta et al. 2022; Kukkar et al. 2022; Mujtaba et al. 2023).

Lignocellulosic biomass has proven to be the most abundant and renewable raw material on Earth. It arises from diverse sources, such as agricultural and forestry residues, dedicated energy crops, and organic fractions from recycling stations (Dutta et al. 2022; Kukkar et al. 2022; Mujtaba et al. 2023). This type of materials are great feedstocks to produce valuable compounds, namely biofuels, biochemicals, bioplastics, among others (Dutta et al. 2022). Although the structural composition of lignocellulose varies according to the type of biomass source, it is essentially composed of cellulose (40–50%), hemicellulose (25–30%), and lignin (15–20%), in addition to carbohydrates, proteins, and extractives (e.g., lipids, resins, terpenes, flavonoids, and phenolic compounds) (Dutta et al. 2022; Kukkar et al. 2022; Mujtaba et al. 2023).

Composting is an eco-friendly and efficient microbiological process through which different organic wastes, such as lignocellulosic residues, are converted into simpler compounds (Sánchez et al. 2017; Li et al. 2023). A typical composting process involves four main phases, i.e., mesophilic, thermophilic, maturation, and curing. In the thermophilic phase, the compost piles reach high temperatures, above 45 °C, due to the intense microbial activity of thermophilic populations that release a wide range of thermostable enzymes able to degrade complex molecules, which are crucial for the lignocellulose degradation (Kukkar et al. 2022; Finore et al. 2023). The synergistic action of a cocktail of enzymes with diversified activities is required for a complete breakdown of lignocellulose into fermentable sugars. This set of enzymes, collectively named carbohydrate-active enzymes (CAZymes), include hemicellulases (e.g., xylanases, mannanases, and arabinofuranosidases) that degrade the polysaccharides present in hemicellulose. Xylanases, namely endo-1,4-β-xylanases (EC 3.2.1.8), are recognized as important mediators in the hydrolytic catalysis of xylan polymers, more specifically in the cleavage of the internal β-1,4-d-xylosidic bonds (Gupta et al. 2022; Kukkar et al. 2022; Mendonça et al. 2023). This type of enzymes is produced by a wide range of microorganisms, namely bacteria and fungi, and has already been exploited in several biotechnological applications, including in the biofuel, pulp and paper, food, feed animal, and textile industries (Ajeje et al. 2021).

Due to their remarkable ability to produce robust lignocellulose-degrading biocatalysts, there is a growing interest in exploring and accessing microbial communities of extreme habitats (e.g., composting). However, these microorganisms and their biomolecules are usually not attainable by conventional cultivation methods (Ajeje et al. 2021). The use of metagenomic approaches contributes to overcoming this issue, as these techniques allow studying the whole microbiota of a particular niche, including microorganisms never before cultivated or difficult to be isolate in pure culture. Metagenomics is a powerful tool to investigate the total genetic material directly sourced from environmental samples, providing a comprehensive taxonomic and functional profile of microbial communities. Mining of novel enzymes with efficient catalytic activities through metagenomics can be performed using two types of approaches, namely sequence- and function-based metagenomics. The sequence-based approach allows the characterization of microbial communities in terms of taxonomy and the prediction of functional diversity by applying complex sequencing methods and databases (e.g., CAZy database) (Kukkar et al. 2022; Santos-Pereira et al. 2023; Finore et al. 2023). On the other hand, function-based metagenomics is focused on the discovery of new metabolic pathways and genes encoding functional enzymes or other bioactive molecules of biotechnological relevance (Pabbathi et al. 2021; Kukkar et al. 2022).

This work reports, for the first time, a functional metagenomic study performed using a compost sample from a Portuguese composting unit aiming to find interesting xylanases. For that purpose, an innovative two-step functional screening based on MATLAB’s in-house functions was developed and a promising clone with xylanase activity (LP4) was identified. By sequencing and taxonomic analysis, the protein (Xyl4) was found to belong to glycosyl hydrolase family 10 (GH10). *xyl4* was subcloned and heterologously expressed in *Escherichia coli*. The three-dimensional (3D) structure of Xyl4 was, for the first time, predicted and refined using in silico tools, and the protein was docked with xylohexaose to study substrate-protein interactions.

## Material and methods

### Strains, plasmid, and reagents

The CopyControl™ Fosmid Library Production kit, including the autoinduction solution (500 ×), and the FosmidMAX™ DNA Purification kit were provided by Epicentre Biotechnologies (Madison, Wisconsin, USA). The NucleoSpin® Gel and PCR Clean-up kit and the NucleoSpin® Plasmid QuickPure™ kit were obtained from Macherey-Nagel (Düren, Germany). Azurin-crosslinked xylan (AZCL-xylan) was supplied by Megazyme (Wicklow, Ireland) and xylan from beechwood was obtained from Carl Roth (Karlsruhe, Germany). Congo red was acquired from HIMEDIA Laboratories Pvt. Ltd. (Maharashtra, India). *Nde*I and *Bam*HI restriction enzymes and T4 DNA ligase were provided by Thermo Fisher Scientific (Waltham, Massachusetts, USA). pETDuet-1 expression vector was supplied by Novogene (Cambridge, UK). *E. coli* NZY5α, *E. coli* BL21 (DE3), bovine serum albumin (BSA), and NZYColour Protein Marker II were delivered by NzyTech (Lisboa, Portugal). Xylanase from *Thermomyces lanuginosus*, lipase from *Candida rugosa*, and other assay reagents used in this work (analytical grade) were purchased from Sigma-Aldrich (St. Louis, IL, USA).

### Sample collection and DNA extraction

Compost sample was obtained from a Portuguese composting unit (LIPOR) located in Baguim do Monte, Porto, Portugal (41°11′58.4″N 8°32′46.6″W), which handles food wastes (40%), green (25%), and forestry (35%) residues. This company follows a well-established composting process, controlling and monitoring the initial residues’ composition and type, processing time, and temperature. The sample was collected at the thermophilic phase of the process (temperature of 50 °C) with 4 weeks of composting (Santos-Pereira et al. 2023). Until DNA extraction, the sample was stored at 4 °C. Metagenomic DNA was extracted from 1 g of compost sample following a protocol developed by our research group, already described by Costa and colleagues (Costa et al. 2021).

### Construction of the compost metagenomic library

CopyControl™ Fosmid Library Production kit was used for the construction of the metagenomic library according to the manufacturer’s instructions. The high molecular weight DNA (≈ 40 kb) isolated from the compost sample (Santos-Pereira et al. 2023) was end-repaired and ligated to the pCC1FOS™ vector. The ligation product was packaged into lambda phage and transformed into *E. coli* EPI300-T1^R^ cells (Epicentre Biotechnologies, Madison, Wisconsin, USA). Transformants were grown overnight at 37 °C on lysogeny broth (LB) agar plates supplemented with 12.5 μg/ml chloramphenicol. A total of 563 transformants were isolated and grown in LB medium supplemented with 10 mM MgSO_4_, 2 g/l maltose, 12.5 μg/ml chloramphenicol, and 0.2% (v/v) CopyControl™ fosmid autoinduction solution (500 ×) at 37 °C. The metagenomic library was stored at − 80 °C in LB medium supplemented with 20% (v/v) of glycerol.

### Functional screening of the metagenomic library to detect xylanase activity

The selection of positive clones for xylanase activity was performed in two steps. Firstly, the activity of all clones of the metagenomic library was evaluated at room temperature (around 25 °C). Afterwards, the positive clones previously identified were incubated at different controlled temperatures (25, 37, 45, and 60 °C) to select the fastest and most promising clones.

#### Screening at room temperature

The functional screening of the metagenomic library was based on a rapid and simple chromogenic screening test to detect xylanase activity at room temperature (approx. 25 °C). The test was performed on 96-well microplates containing a suitable substrate (AZCL-xylan) for this enzymatic activity. After the growth of the 563 clones in LB medium supplemented with 12.5 μg/ml chloramphenicol, 10 mM MgSO_4_, 2 g/l maltose, and 0.2% (v/v) CopyControl™ fosmid autoinduction solution (500 ×), they were transferred to 96-well microplates with LB agar, 12.5 μg/ml chloramphenicol, 10 g/l arabinose, and 0.05% (w/v) AZCL-xylan. After incubation overnight at 37 °C for microbial growth, the 96-well microplates were kept at room temperature for 1 week. The appearance of the blue color was considered a positive response, as previously reported by Knapik et al. (2019). Commercial enzymes, namely xylanase from *T. lanuginosus* and lipase from *C. rugosa*, were used as positive and negative controls, respectively.

#### Screening at controlled temperatures (25, 37, 45, and 60 °C)

Besides the room temperature (25 °C), other temperatures were studied in the second step of the screening test. The temperature of 37 °C was included since it is the optimal temperature for the host microbial growth (*E. coli*). On the other hand, 45 and 60 °C were selected as temperatures close to the sampling temperature (50 °C). All the xylanase positive clones previously identified at room temperature (12 clones), together with eight clones with negative response randomly selected, were submitted to a new functional screening at different controlled temperatures (25, 37, 45, and 60 °C) with the above-mentioned substrate and culture medium in Petri plates. To avoid undesirable agar cracks, temperatures above 60 °C were not tested. Initially, the clones were grown overnight, and their xylanase activity was evaluated after 1 and 4 days of incubation. The presence of blue color was assumed as a positive response and the size of the halo areas was determined.

### Estimation of xylanase activity based on blue halo area measurement

The Petri plates were divided into equal squares to delimitate the individual areas to be screened. The existence and increasing area of the blue color were qualitatively correlated with an increase positive response. A pipeline to process both the acquisition of the images and the estimation of the blue halo area was devised.

#### Image acquisition

**Fig. 1:**
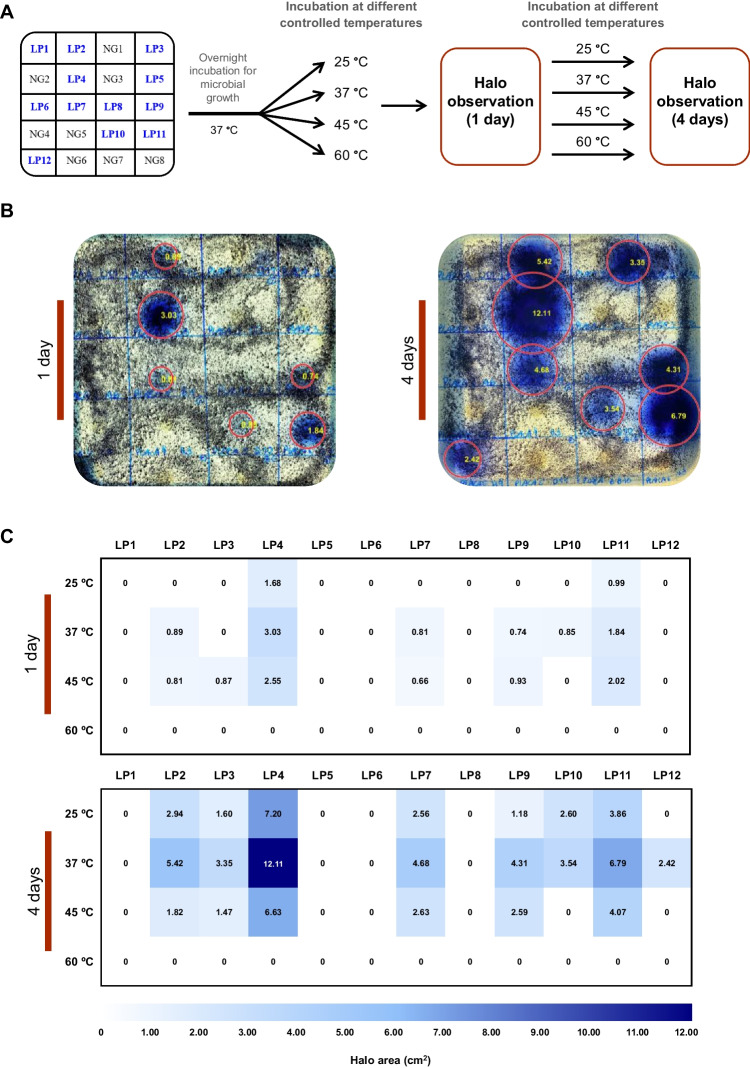
Functional screening in Petri plates at different controlled temperatures (25, 37, 45, and 60 °C) to select the most promising clone with xylanase activity by analyzing color images using MATLAB’s in-house functions. **A** Division of the Petri plates into squares to delimitate the analysis area of the 12 positive clones (LP1 to LP12, in blue) and 8 negative clones (NG1 to NG8, in black) evaluated for xylanase activity at different incubation temperatures after overnight growth at 37 °C. The measurement of the blue halos areas was performed after 1 and 4 days of incubation. **B** Example of the visual depiction of the results obtained after 1 and 4 days of incubation at 37 °C according to the clone distribution represented in **A**. The red circles delimit the areas of the blue halos extracted automatically using MATLAB’s in-house functions. **C** Representation in a density matrix of the halo areas, in cm^2^, extracted from each individual square corresponding to a positive clone (LP1 to LP12), inoculated as denoted in A. All the extracted areas after 1 and 4 days of incubation at different controlled temperatures (25, 37, 45, and 60 °C) were included. An increase in the blue color saturation was directly correlated with the size of the halo area and with a positive response

Each Petri plate was back illuminated with a cold LED lamp (Aigostar, Toledo, Spain) to provide uniform or close to uniform background lighting to minimize the spatial non-uniformities produced by shadows or areas with diminished illumination to improve the image acquired. Images were acquired using a mobile phone (iPhone 11, Apple Inc, Cupertino, California, USA) placed on a fixed support on top of the Petri plate. The imaging resolution of the mobile phone was set to maximum; the photo settings as the exposure time, white balancing, assumed illuminant, and the aperture were set to automatic; and the flash was turned off, so no other illumination source apart from the lamp placed at the back of the Petri plate was used. Images provided by the software of the mobile phone were retrieved and transferred into a computer for analysis with no additional or intended image processing. Resulting images of such acquisition can be seen in Fig. 1B, as an example.

#### Image processing

All computations were done using MATLAB and Image Processing Toolbox (MATLAB R2022a, MathWorks, Natick, MA, USA).

Images of each Petri plate compressed for the sRGB color space, as retrieved from the mobile phone, were converted into the CIELAB color space assuming the CIE 2° 1931 color vision observer and the CIE D65 illuminant. Such color space codes color as lightness (L*) ranging from 0 to 100, green–red colors (a*) ranging from − 100 to 0 for green and 0 to + 100 for red, and blue-yellow colors (b*) ranging from − 100 to 0 for blue and 0 to + 100 to yellow. As the experimental positive response was correlated with the intensity of the blue color, negative values of b* were considered positive responses, assuming values from − 20 < b* <  − 5, with more negative values being bluer than values less negative. The use of such color space enabled a more precise selection, individualization, and segmentation of the blue component in each image. An automated script was created to identify rounded areas (halo areas) of high density of negative b* component, extracting the number of pixels on each halo area selected. If the automated extraction was not capable of providing the number of pixels, a manual script was used. The manual script overimposed a round mask over the image, enabling the adjustment of the mask size and position to the halo area. Visual adjustments to the size and position of the masks were performed manually assuming the same constraints as the ones for the automated script, and the number of selected pixels occupied was extracted. The same criteria to select the manual mask were used across all trials.

#### Estimating the area size

The size of each pixel was estimated for each processed image so an estimation of the area occupied by the halo area could be derived. A line was drawn on top of each image, with adjustable position and size. The line was drawn to match the pixel distance between two consecutive vertical blue lines, and the number of pixels of the line was retrieved. The physical distance between the blue lines was then measured and made to match the retrieved number of pixels, establishing the physical size to the retrieved number of pixels and hence the size in millimeters of each pixel and its corresponding area. The area in square centimeters of each rounded mask was then computed by counting the number of pixels.

To estimate the effect of the temperature and time on the size of the halo area, a comparison was made by representing the area of each individual square into a density matrix. An increase in the blue color saturation was directly correlated with the size of the halo area, extracted from acquired images, and directly correlated with a positive response. All computations were done using MATLAB and Image Processing Toolbox (MATLAB R2022a, MathWorks, Natick, MA, USA).

### Validation of LP4 clone as xylanase producer

#### Production of the crude enzymatic extract

The selected positive clone (LP4) was grown in LB medium supplemented with 12.5 μg/ml chloramphenicol, 10 mM MgSO_4_, and 2 g/l maltose at 37 °C at 200 rpm. Additionally, *E. coli* EPI300-T1^R^, the microbial host previously used to prepare the metagenomic library, was grown under the same conditions, but in a medium without the antibiotic, to serve as negative control in the enzymatic experiments. When OD_600_ of the cultures reached 0.7, enzyme expression was induced using 0.2% (v/v) CopyControl™ fosmid autoinduction solution (500 ×). The cells were harvested 20 h post-induction by centrifugation at 9000 × *g* for 5 min. For extracellular activity, the supernatant was concentrated using Vivaspin® 20 centrifugal concentrators with a 10-kDa cutoff membrane (Sartorius AG, Göttingen, Germany) according to the manufacturer’s instructions. The cells were resuspended in 1 ml lysis buffer (0.6 M mannitol, 2 mM EGTA, 10 mM Tris, 1 × protease inhibitor cocktail, pH 6.8) and the cell disruption was carried out with glass beads (425–600 µm; approximately one-third of the tube volume) on a cellular disruptor (FASTprep-24, MP Biomedicals, Irvine, CA, USA). Cell disruption occurred at a speed setting of 6.5 m/s for 3 cycles for 60 s. Cellular debris was pelleted by centrifugation at 13000 × *g* for 10 min at 4 °C, and the cell lysate (supernatant) was collected to test the intracellular activity. The total protein concentration was determined by the Bradford assay method, using BSA solutions of known concentration as standards, and by measuring the absorbance at 595 nm (Bradford 1976).

The concentrated supernatants (extracellular fractions) and the cell lysates (intracellular fractions) were stored at − 20 °C.

#### Enzymatic assays for xylanase activity determination

The ability of the extracellular and intracellular fractions to degrade commercial beechwood xylan was determined by measuring the concentration of reducing sugars using the dinitrosalicylic acid (DNS) method (Miller 1959). In all conditions, the enzyme reaction mixtures contained 50 µl of 1% (w/v) beechwood xylan dissolved in 62 mM Tris-HCl with a pH value of 6.8 and 50 µl of enzyme fraction (extra or intracellular). Mixtures were left to react at 50 ℃ for 30 min, and the reaction was stopped by the addition of 100 µl DNS reagent solution, and further incubation at 95 ℃ for 5 min. Then, the enzymatic mixtures were cooled on ice and 1 ml of distillated water was added. The amount of reducing sugars released from the hydrolysis of commercial beechwood xylan was determined by measuring the absorbance at 540 nm using xylose as standard. One unit (U) of enzyme activity is defined as the amount of enzyme required to release 1 mmol of xylose from commercial beechwood xylan per min under the assay conditions.

#### Zymography

Zymogram analysis for xylanase was performed by incorporating 1% (w/v) of commercial beechwood xylan into 10% non-denaturing polyacrylamide gel. The samples were previously diluted (1.5 µg of protein for extracellular fraction and 40 µg of protein for intracellular fraction) with 62 mM Tris–HCl buffer (pH 6.8) containing 20% (v/v) glycerol and 0.02% (w/v) bromophenol blue. The gel was run at 4 °C, for 3 h under constant amperage 20 mA. Then, the gel was incubated at 37 °C in 0.1 M sodium acetate buffer at pH 6.5 for 1 h. After that, it was stained with 0.1% (w/v) Congo red, and finally distained with 1 M NaCl to detect the functional activity of the crude enzymatic extracts (adapted from Pavarina et al. 2021). The positive response for xylanase activity was shown as clear hydrolysis bands against the dark red background. 2.5 U of commercial xylanase from *T. lanuginosus* was used as positive control.

### Fosmid sequencing, assembly, insert identification, and phylogenetic analysis

Raw sequence reads were obtained with paired-end short-read (150 bp) sequencing, using the Illumina NovaSeq6000 Platform at Eurofins Genomics (Ebersberg, Germany), and checked for quality using FastQC software v. 0. 11.9 (https://www.bioinformatics.babraham.ac.uk/projects/fastqc/). Clean reads were assembled into contigs using MEGAHIT v.1.2.9 (Li et al. 2015), using standard parameters, and Quast software v.5.2.0 (Gurevich et al. 2013) was used to assess assembly quality. The DNA sequence of the cloning vector (accession EU140751—cloning vector pCC1FOS™) was used as query, using BLASTn (Basic Local Alignment Search Tool) v. 2.13.0, locally, against the assemblies. Results were used to extract the insert DNA sequences. ORFfinder (Wheeler et al. 2003) was used to search for open reading frames (ORFs) in the insert sequence, resulting in a total of 25 possible sequences. Afterward, eggNOG-mapper v.2.1.9 (Cantalapiedra et al. 2021) was used to functionally annotate the obtained ORFs, and choosing orthologs that were inferred from experimental evidence. Gene function predictions were also accomplished by assessing the CAZymes database (Cantarel et al. 2009). Based on these results, three ORFs were selected for further analyses based on their annotation as glycosyl hydrolases (GH). A blast analysis was then performed using BLASTp (https://blast.ncbi.nlm.nih.gov/) against the non-redundant protein sequences (nr) database to identify the sequence coding for a xylanase. This sequence was next submitted to a second round of BLAST analysis, being identified using the EMBL-EBI AlphaFold database (https://www.ebi.ac.uk/Tools/sss/fasta/). In particular, the first 50 FASTA sequences were collected and multiple-aligned using ClustalW in MEGA11 software (Tamura et al. 2021), using standard parameters. Following the alignments, a neighbor-joining tree was built in MEGA11, using standard parameters. iTOL (Letunic and Gmbh 2021) was used to visualize and edit the tree. The molecular mass of the encoded protein was predicted by ApE (ApE Plasmid Editor, version 2.0.49 by Davis and Jorgensen (2022)).

### Expression of the recombinant xylanase

#### Subcloning

The fosmid DNA from the positive clone (LP4) was recovered using a FosmidMAX™ DNA Purification kit. The full xylanase gene (*xyl4*) was amplified by PCR using the following primer pair: *xyl4_nde*i*_fw* 5′-cccCATATGAGCATTTCCCGTCGTAA-3′ (containing restriction site for *Nde*I) and *xyl4_bam*hi*_rev* 5′-ggGGATCCTTAACGTTTTAAATTCATCAATCG-3′ (containing restriction site for *Bam*HI). The amplified product was purified with NucleoSpin® Gel and PCR Clean-up kit. After digestion with *Nde*I and *Bam*HI restriction enzymes for 1 h at 37 °C, the pETDuet-1 expression vector and PCR product were ligated using the T4 DNA ligase enzyme for 1 h at 22 °C, constructing the recombinant plasmid pETDuet-1_*xyl4*. The transformation of the pETDuet-1_*xyl4* plasmid was performed into host competent cells *E. coli* NZY5α. The positive transformant colonies were selected based on ampicillin resistance and the pETDuet-1_*xyl4* plasmid was isolated using the NucleoSpin® Plasmid QuickPure™ kit. Finally, for the expression of the xylanase gene, the pETDuet-1_*xyl4* plasmid was transformed into *E. coli* BL21(DE3) originating the Xyl4 strain.

#### Evaluation of the xylanase activity

The transformant was grown in LB medium containing 100 μg/ml ampicillin at 37 °C. As control, the *E. coli* BL21(DE3) wild type was grown under the same conditions, but in a medium without antibiotic. When OD_600_ of the culture reached 0.6, the expression of the recombinant gene was induced by the addition of isopropyl β-d-1-thiogalactopyranoside (IPTG) at two concentrations, namely 0.1 and 1 mM. After 16 h at 30 °C, the cells were harvested by centrifugation at 9 000 × *g* for 5 min. The cells were treated as previously mentioned in section dedicated to the production of the crude enzymatic extract. The cell lysates (intracellular fractions) were stored at − 20 °C.

The concentration of the recombinant protein was determined by the Bradford assay method using BSA as standard, as aforementioned. The molecular weight of the enzyme was estimated by 12% sodium dodecyl sulfate polyacrylamide gel electrophoresis (SDS-PAGE) using a standard protein marker (NZYColour Protein Marker II). As described in the section dedicated to enzymatic assays for xylanase activity determination, the enzyme activity in the cell lysates (intracellular fraction) was quantified by the DNS method. In addition, the xylanase activity was studied through the zymogram technique.

### Study of xylanase-substrate interaction

#### Prediction of enzyme 3D structure, model preparation, and refinement

The amino acid sequence of the protein was submitted to AlphaFold2 (Jumper et al. 2021) for 3D structure prediction. The top prediction was then refined by molecular dynamics (MD) simulations using the AMBER software, with the protein treated with ff14SB force field (Maier et al. 2015), embedded in a box of TIP3P water molecules with a minimum distance of 12 Å between the protein and the box side, and neutralized by counter ions with Leap (Case et al. 2005). Energy minimization steps were applied to remove clashes, followed by two equilibration steps and a final production run. The minimization steps were applied to the following groups of atoms: first minimization, water molecules (2500 steps); second minimization, hydrogens atoms (2500 steps); third minimization, chains of all the amino acid residues (2500 steps); forth minimization, full system (10000 steps). The two 50 ps equilibration steps consisted of (i) heating of the system to 298 K using a Langevin thermostat at constant volume (NVT ensemble) and (ii) equilibration of the density of the system at 298 K. Lastly, the 100-ns production run was performed in an NPT ensemble with a temperature of 298 K and 1 bar pressure. Visual molecular dynamics (VMD) (Humphrey et al. 1996) and the cpptraj tool (Roe and Cheatham 2013) were employed to explore the resulting MD trajectories. The MD simulations were performed under periodic boundary conditions to simulate a continuous system. The SHAKE algorithm was used to fix all the bond lengths involving hydrogen atoms, together with an integration step of 2 fs.

#### Molecular docking

A representative structure of the dominant conformation adopted by the protein along the MD simulation was chosen for the molecular docking stage. This selection was performed through cluster analysis using the K-means clustering method to evaluate all conformations recorded during the MD simulation, enabling the identification of 5 main clusters illustrating the range of conformations that the protein can adopt in solution. These clusters were obtained using the root mean square deviation (RMSD) of all non-hydrogen atoms as a similarity measure. To predict the binding conformation and affinity of a xylose-based substrate, the protein-ligand docking program GOLD was used (Jones et al. 1997). Since the xylan sugar chains are considered too long for molecular docking, the model substrate chosen as example for evaluation was xylohexaose (Xie et al. 2021). A docking region with a 30 Å radius centered around the putative catalytic residues Glu133, Glu238, and His209 was considered. Docking was performed with the ChemPLP for a total of 500 GA runs per ligand.

#### Molecular dynamics simulations and free energy calculations

To validate the docking predictions, MD simulations were performed with the most stable enzyme-xylohexaose predictions from docking. The substrate was parameterized with Gaussian16 (Frisch et al. 2016) using ANTECHAMBER and the General Amber Force Field (GAFF) (Wang et al. 2004, 2006) with RESP HF/6-31G(d) charges. These simulations were extended to 1000 ns using the same protocol described for the enzyme alone (section “prediction of enzyme 3D structure, model preparation, and refinement”). The free energy method MM-GBSA (molecular mechanics-generalized born surface area method) was employed to estimate the binding free energy of the substrate to the enzyme. For that purpose, the MM/PBSA.py script (Miller et al. 2012), available in AMBER, was applied, with a salt concentration of 0.100 mol dm^−3^, considering 1000 frames per complex. The free energy decomposition option was used to obtain information about the contribution of each residue to the total free energy.

### Statistical analysis

All experiments were carried out in triplicate and analyzed using the GraphPad Prism software version 8.0.1 (GraphPad Software, Inc., San Diego, CA, USA). All the data are presented as mean values ± standard deviation. The statistical significance and differences were evaluated by one-way ANOVA with Tukey’s multiple comparison test when *p* < 0.05.

## Results

### Identification of xylanases by functional metagenomics

The composting units handling lignocellulosic materials are suitable sources for finding novel and interesting industrial biocatalysts, namely xylanases. A metagenomic fosmid library containing 563 clones was constructed using DNA extracted from a compost sample. In the first functional screening, all the clones were screened for xylan-active enzymes. After 1 week at room temperature, 12 out of the 563 clones (2.1%) showed a positive response indicated by the appearance of blue color in the wells.

The 12 positive clones, together with 8 randomly selected negative clones (negative controls), were submitted to a new functional screening in Petri plates under controlled temperatures to select the most promising clone for further studies (Fig. 1A). While for some clones a positive response (blue halo areas) was detected, for others no color changes were observed (no or reduced/fainted blue halo areas) (Fig. 1B). As expected, no color change was detected in the negative controls. As shown in Fig. 1C, it was found that a temperature of 60 °C was not favorable to detect xylanase activity, since it was not possible to extract any blue halo area. Furthermore, some technical issues, such as the dryness of the culture medium, were detected at 60 °C, leading to the formation of some agar cracks. In addition, it was confirmed that in the screening performed at different controlled temperatures for a shorter period, the LP1, LP5, LP6, and LP8 clones did not show a positive response, contrary to the results found in the previous screening (1 week). The highest xylanase activity was detected at 37 °C (Fig. 1B), as halos of higher area were found. The assessment of the halo area after 1 and 4 days of incubation was coincident and support this claim (Fig. 1C). Noteworthy, after 1 day of incubation, the LP4 and LP11 clones were the only ones that showed enzymatic activity at 25, 37, and 45 °C (Fig. 1C). Considering both the faster xylanase activity at different temperatures and the development of larger blue halos, the LP4 clone was selected to be further validated and evaluated as a xylanase producer.

### Evaluation of the LP4 clone xylanase activity

**Fig. 2:**
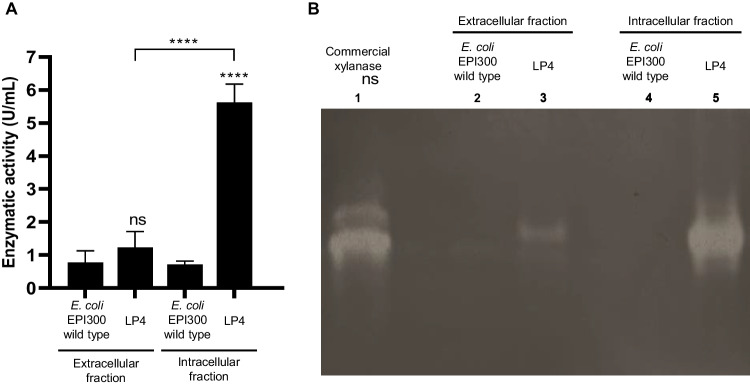
Evaluation of the xylanase activity of the LP4 clone in the extracellular and intracellular fractions. **A** Enzymatic activity (U/ml) of the LP4 clone compared to the *E. coli* EPI300, which was used as negative control. Results are the triplicate analysis of each sample ± standard deviation. ****p < 0.0001 compared to the corresponding cell fraction of the negative control and by comparison of the two cell fractions of the clone under study. **B** Zymogram analysis of the LP4 clone on a 1% (w/v) beechwood xylan-complemented NATIVE PAGE gel compared to the *E. coli* EPI300, which was used as negative control. Clear areas in the zymogram indicate enzymatic activity. Lane 1—commercial xylanase from *T. lanuginosus*; lane 2—extracellular fraction of the control (wild-type strain); lane 3—extracellular fraction of the LP4 clone; lane 4—intracellular fraction of the control (wild-type strain); lane 5—intracellular fraction of the LP4 clone

The selected LP4 clone was then evaluated as an effective producer of a xylanase under submerged fermentation conditions. The xylanase activity of the clone was quantified by the DNS method and assessed by zymogram analysis using commercial beechwood xylan (Fig. 2). The enzymatic activity of the extracellular and intracellular fractions was estimated (Fig. 2A). Compared to the control (non-transformed host strain), the LP4 clone showed a much higher enzymatic activity in the intracellular fraction, which confirmed the ability of this clone to produce a xylanase. In the extracellular fraction, 1.23 ± 0.39 U/ml of crude enzyme was obtained. Nevertheless, there were no significant differences for this fraction when compared with the control. In the intracellular fraction, the enzymatic activity achieved was 5.62 ± 0.45 U/ml of crude enzyme, exhibiting significant differences in relation to the control. Additionally, a clear area was observed in the zymogram in both fractions (Fig. 2B, lanes 3 and 5), confirming the presence of an active form of xylanase.

### Identification of xylanase sequence and phylogenetic analysis

To identify the xylanase sequence and proceed with its subcloning, the respective fosmid was extracted and sequenced. After the assembly, the blast against the pCC1FOS™ fosmid sequence, and the analysis with the ORF finder, a 1137 bp ORF (GenBank accession number PP171485) was predicted to encode a protein comprising 378 amino acids with a calculated molecular mass of 42.8 kDa. EMBL-EBI Blast and mapping against the Eggnog database identified this protein as an endo-1,4-β-xylanase belonging to the GH10 family.

**Fig. 3:**
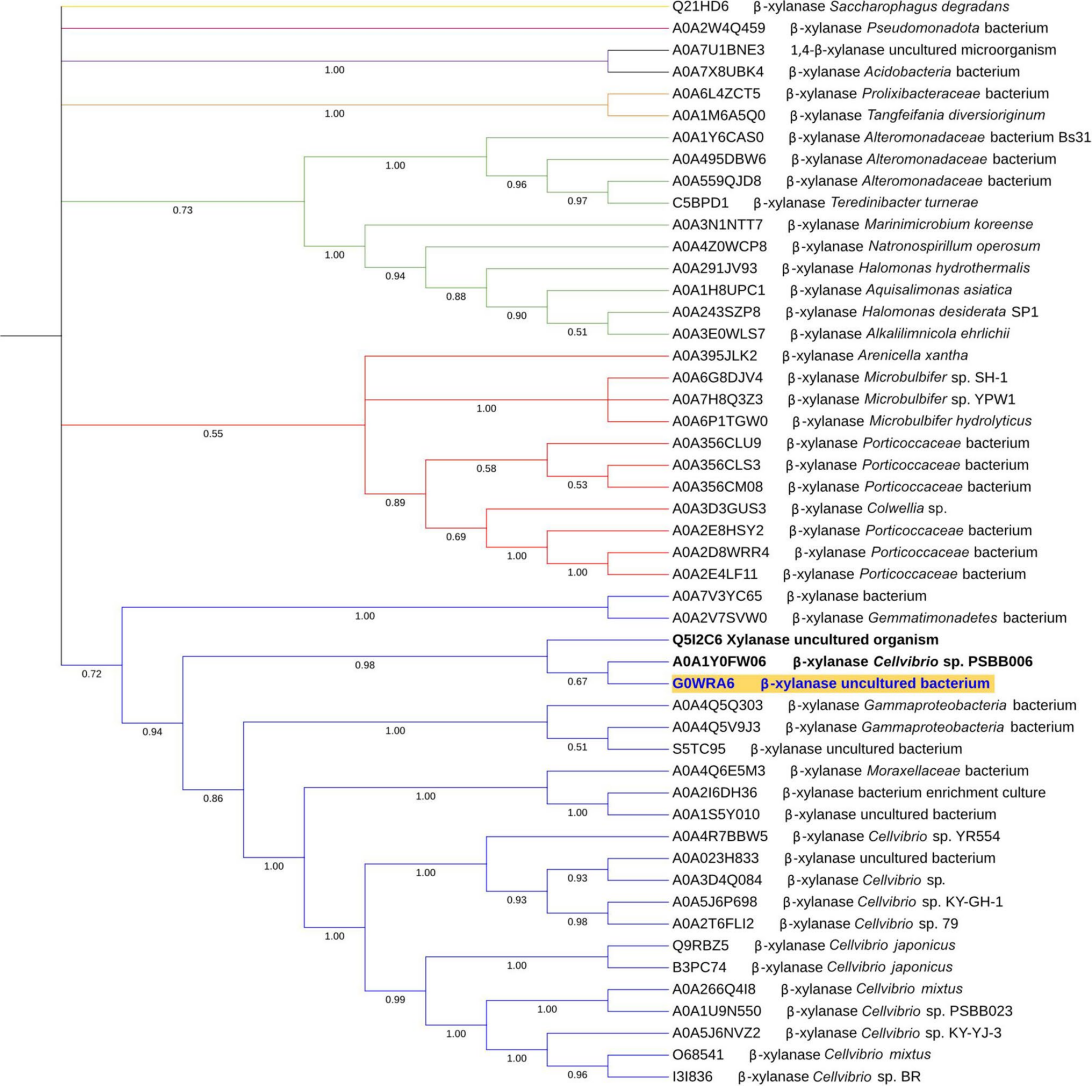
Phylogenetic analysis based on the amino acid sequence of the endo-1,4-β-xylanase identified in this study (marked yellow) and the 50 most related β-xylanases using the neighbor-joining method. Phylogenetic analysis was performed using ClustalW and MEGA1 (Tamura et al. 2021). Numbers below branches represent bootstrap values

The cladogram of the Fig. 3, obtained by neighbor-joining analysis, shows the clustering of the 50 β-xylanases classified as being the most similar to the endo-1,4-β-xylanase identified in this study, as obtained by EMBL-EBI Blast. Protein G0WRA6 (marked as yellow in cladogram) (Jeong et al. 2012) yielded 100% identity with an e-value of 1.0E^−159^ to the enzyme identified in this study, being located in close similarity with the β-xylanase of *Cellvibrio* sp. PSBB006, and with a xylanase from an uncultured organism—Q5I2C6 (bold in cladogram).

The cladogram reveals the existence of three major clusters, separated from six other sequences that do not cluster with them. The first major cluster (blue), containing the sequences most closely related to the xylanase herein discovered, includes mainly β-xylanases of *Cellvibrio* species, while the red cluster is composed mainly of β-xylanases of *Porticoccaceae* and *Microbulbifer* bacteria, both clusters corresponding to organisms of the order *Cellvibrionales*. Green cluster, even though composed of proteins also from *Cellvibrionales* (*Teredinibacter* and *Macrinimicrobium*), contains, however, β-xylanases from bacteria of other orders, particularly from *Alteromonadales* (*Alteromonadaceae*), *Oceanospirillales* (*Natronosporillum* and *Halomonas*), and *Chromatiales* (*Aquisalimonas* and *Alkalimnicola*). Sequences outside these three main clusters correspond mainly to β-xylanases from classes other than *Gammaproteobacteria*.

### Recombinant xylanase production

**Fig. 4:**
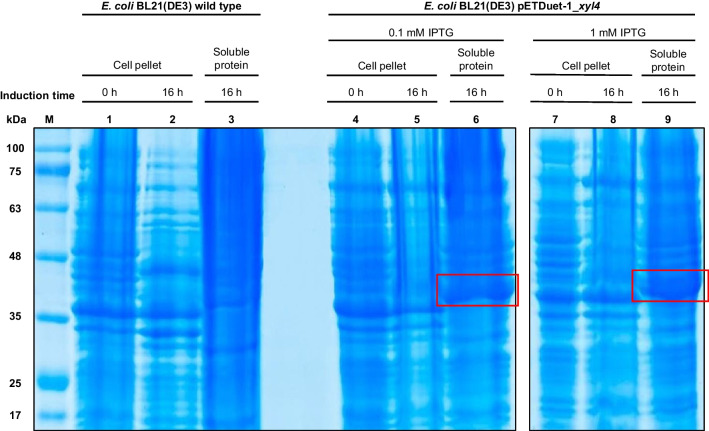
SDS-PAGE analysis of the crude extract of the pETDuet-1_*xyl4* expressed in *E. coli* BL21 (DE3). Lane M—molecular weight marker; lane 1—cell pellet of the *E. coli* BL21(DE3) wild type before induction and cell disruption; lane 2—cell pellet of the *E. coli* BL21(DE3) wild type 16 h after induction and before cell disruption; lane 3—soluble protein of the *E. coli* BL21(DE3) wild type 16 h after induction; lane 4—cell pellet of the pETDuet-1_*xyl4* before induction with 0.1 mM IPTG and cell disruption; lane 5—cell pellet of the pETDuet-1_*xyl4* 16 h after induction with 0.1 mM IPTG and before cell disruption; lane 6—soluble protein of the pETDuet-1_*xyl4* 16 h after induction with 0.1 mM IPTG; lane 7—cell pellet of the pETDuet-1_*xyl4* before induction with 1 mM IPTG and cell disruption; lane 8—cell pellet of the pETDuet-1_*xyl4* 16 h after induction with 1 mM IPTG and before cell disruption; lane 9—soluble protein of the pETDuet-1_*xyl4* 16 h after induction with 1 mM IPTG

Recombinant enzyme expression into *E. coli* was carried out aiming to increase xylanase production and improve enzyme activity. For that, pETDuet-1_*xyl4* was produced in *E. coli* BL21(DE3) and only the intracellular enzyme was recovered since this fraction showed higher activity in the previous tests. The crude enzyme was analyzed by SDS-PAGE (Fig. 4) by comparison with the crude extract of the *E. coli* BL21(DE3) without the pETDuet-1_*xyl4* plasmid. A more intense and differentiating band between 35 and 48 kDa (delimited in red in Fig. 4) was visible in the soluble fractions of both crude enzymatic extracts of the Xyl4 strain induced with different concentrations of IPTG, 1 mM (lane 6) and 0.1 mM (lane 9), which agrees with the predicted molecular weight of the recombinant enzyme (≈ 43 kDa).

**Fig. 5:**
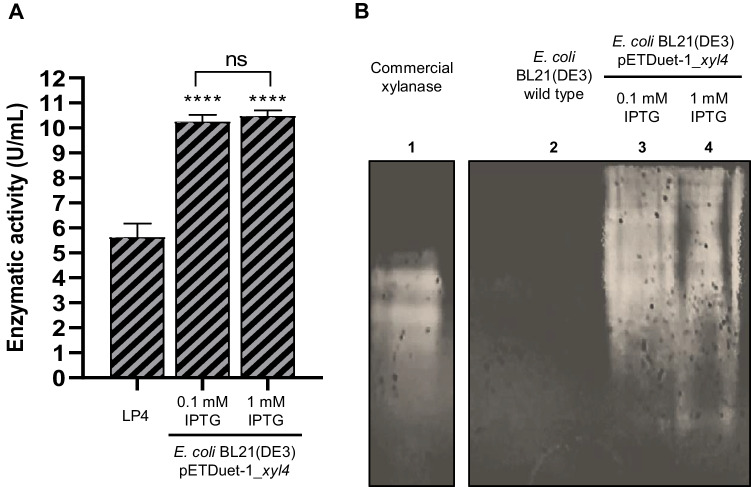
Evaluation of the xylanase activity of the intracellular crude extracts of *E. coli* cells expressing the pETDuet_*xyl4* (Xyl4 strain). **A** Enzymatic activity (U/ml) of Xyl4 compared to the LP4 clone (same data as in Fig. 2A). Results are the triplicate analysis of each sample ± standard deviation. ****p < 0.0001 by comparison of the intracellular fraction of the Xyl4 strain induced with different IPTG concentrations (0.1 and 1 mM) with the intracellular fraction of the LP4 clone. ns—non-significant when compare enzyme activity after induction with the two different doses of IPTG. **B** Zymogram analysis of Xyl4 on a 1% (w/v) beechwood xylan-complemented NATIVE PAGE gel compared to the *E. coli* BL21 (DE3), which was used as negative control. Clear areas in the zymogram indicate enzyme activity. Lane 1—commercial xylanase from *T. lanuginosus*; lane 2—intracellular fraction of the negative control; lane 3—intracellular fraction of Xyl4 strain using 0.1 mM IPTG; lane 4—intracellular fraction of Xyl4 strain using 1 mM IPTG

Regarding the catalytic activity analyzed using the commercial beechwood xylan, significantly higher values were obtained for the intracellular fraction of the crude enzymatic extracts of the Xyl4 strain induced with different concentrations of IPTG, when compared to the intracellular fraction of the LP4 clone (Fig. 5A). However, a higher concentration of IPTG did not result in the increase of the enzyme activity. When the recombinant strain was induced with 0.1 mM IPTG, an enzymatic activity of 10.24 ± 0.23 U/ml of crude extract was detected. Similarly, when induced with 1 mM IPTG, Xyl4 showed an enzymatic activity of 10.48 ± 0.18 U/ml of crude extract (Fig. 5A). Additionally, zymography analysis showed a smeared band for both crude enzymatic extracts of the recombinant strain induced with 0.1 mM and 1 mM IPTG (Fig. 5B). Comparatively with Fig. 2B, Xyl4 showed increased levels of hydrolytic activity, since the amount of protein applied in both tests was the same and the zymogram profile was visibly strengthened (Fig. 5B).

### Prediction of Xyl4 structure and interaction with the substrate

**Fig. 6:**
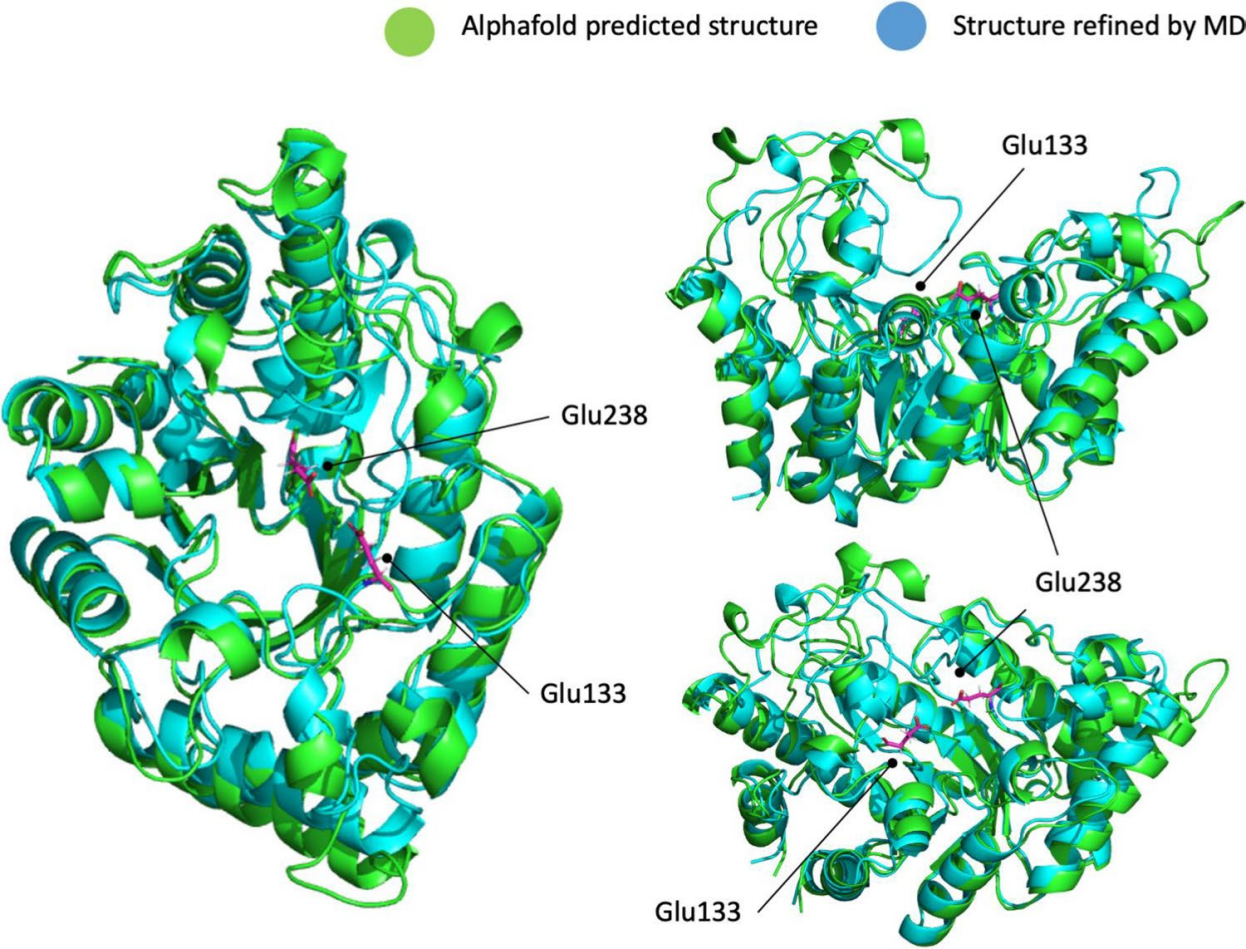
Comparison of the structure of Xyl4 predicted by Alphafold and refined by the MD simulations. The putative catalytic Glu133 and Glu238 residues are highlighted in pink

**Fig. 7:**
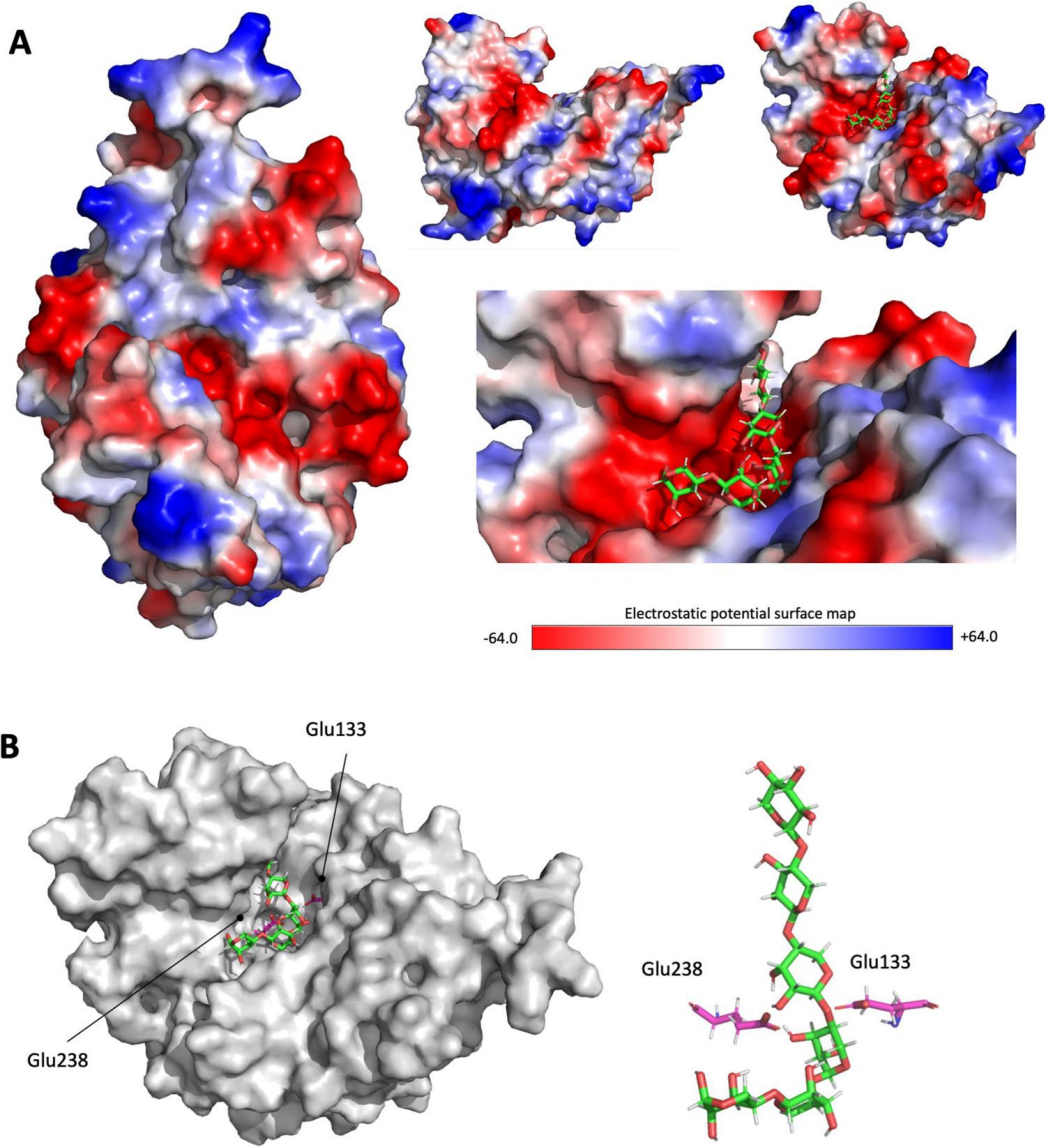
Predicted binding between Xyl4 and xylohexaose and relative position of the putative catalytic residues. **A** Electrostatic potential maps for the enzyme with emphasis on the binding pocket region, highlighting the docked pose of xylohexaose (shown in sticks with the carbon atom represented in green and red for the oxygen atom). The blue color stems for positive potential, white color for neutral potential, and red for negative potential. **B** Binding pose predicted for xylohexaose, highlighting the relative position of the putative catalytic residues

We next sought to predict the 3D structure of Xyl4 and study its interaction with xylohexaose. Based on the previously determined sequence, the structure was predicted using AlphaFold (Fig. 7, in green), followed by further refinement through MD simulations (Fig. 6, in blue). The results show that the MD simulations retain the overall fold and topology of the AlphaFold prediction, and that is maintained at 298 K along the time. The structures reveal the presence of two amino acid residues—Glu133 and Glu238—in close proximity and in opposing directions that could act as catalytic residues, as in other xylanases (Mendonça et al. 2023).

With the refined structure of Xyl4, docking with the GOLD software was performed to analyze a possible interaction with the xylohexaose substrate, using three independent scoring functions (ChemPLP, Astex Statistical Potential (ASP), and Chemscore). The docking scores obtained with the ChemPLP, ASP, and Chemscore scoring functions were 84.6, 70.2, and 62.5, respectively. The different scoring functions available in GOLD evaluate protein-ligand binding using scores that are non-dimensional, with higher values indicating stronger binding.

Figure 7A shows the electrostatic potential maps of Xyl4, also illustrating the relative position of the putative catalytic glutamate residues, further illustrated in Fig. 7B. As shown in Fig. 7A (left), the structure predicted with AlphaFold and refined by MD clearly shows a long cleft, which exhibits a markedly negative electrostatic potential and is comprised by several subsites that accommodate the different xylose moieties of the substrate.

To further clarify the interaction of Xyl4 with the substrate, to confirm the docking predictions, and to measure binding affinity, molecular dynamics simulations and free energy calculations were performed on the enzyme-substrate complexes, taking the dynamic nature of the interaction into account. Table 1 presents the binding free energy values for xylohexaose, as determined based on 1000 conformations obtained along 1000 ns of MD simulation of the possible conformation obtained from docking, capturing the dynamic nature of the enzyme-substrate association and the explicit role of the solvent on the range of conformations adopted. The results show that xylohexaose remains strongly bound to the enzyme with a binding free energy of − 34.5 kcal/mol. Despite the high unfavorable contribution of the polar solvation free energy, arising from the desolvation penalty associated to xylohexaose (71.0 kcal/mol), binding to the enzyme is compensated by the high contribution from van der Waals (− 58.1 kcal/mol) and electrostatic (− 40.1 kcal/mol) interactions.

**Table 1:** Xylohexaose binding free energy values calculated with MM-GBSA and contribution from the different energy terms

	DG binding (kcal/mol)	Contribution to binding free energy (kcal/mol)			
		van der Waals	Electrostatic	Polar component to the solvation free energy	Non-polar component to the solvation free energy
Xylohexaose	− 34.5 ± 0.3	− 58.1 ± 0.3	− 40.1 ± 0.5	71.0 ± 0.4	− 7.3 ± 0.1

Table 2 presents the contribution to the xylohexaose binding free energy of the catalytic amino acid residues (Glu133 and Glu238) and for all amino acid residues with a contribution stronger than − 0.8 kcal/mol (i.e., more negative). The results show that in general, these two catalytic amino acid residues have a neglectable contribution to substrate binding, with their importance likely residing only in catalysis. Among the other amino acid residues, the most important are Trp312, Trp248, Phe316, and His209 with contribution to the binding free energy of − 4.3, − 3.1, − 2.5, and − 2.0 kcal/mol, respectively.

**Table 2:** Xyl4 amino acid residues with highest contribution to xylohexaose binding as calculated with MM-GBSA and contribution from the different energy terms

	Contribution to DG binding (kcal/mol)	Contribution to binding free energy (kcal/mol)			
		van der Waals	Electrostatic	Polar component to the solvation free energy	Non-polar component to the solvation free energy
GLU133	0.9 ± 0.4	− 0.3 ± 0.1	0.9 ± 2.5	0.3 ± 2.5	− 0.1 ± 0.0
TYR176	− 1.3 ± 0.6	− 1.5 ± 0.4	− 0.3 ± 0.8	0.7 ± 0.6	− 0.2 ± 0.1
HIS209	− 2.0 ± 1.2	− 3.1 ± 1.0	− 1.4 ± 1.5	2.9 ± 1.5	-0.4 ± 0.1
GLU238	0.9 ± 0.6	− 0.2 ± 0.1	0.2 ± 2.0	1.0 ± 1.9	0.0 ± 0.0
TRP248	− 3.1 ± 1.0	− 2.9 ± 0.7	− 0.8 ± 0.6	1.0 ± 0.4	− 0.4 ± 0.1
ILE255	− 1.0 ± 0.9	− 1.0 ± 0.9	0.0 ± 0.7	0.2 ± 0.6	− 0.2 ± 0.2
ARG258	− 0.8 ± 1.8	− 0.8 ± 1.1	− 3.4 ± 5.7	3.5 ± 5.1	− 0.1 ± 0.2
TRP304	− 0.9 ± 0.8	− 1.1 ± 0.7	− 0.1 ± 0.5	0.5 ± 0.4	− 0.2 ± 0.1
SER311	− 1.3 ± 1.3	− 0.6 ± 0.6	− 0.9 ± 1.7	0.3 ± 0.6	0.0 ± 0.0
TRP312	− 4.3 ± 1.8	− 4.4 ± 1.4	− 0.7 ± 0.8	1.2 ± 0.5	− 0.4 ± 0.1
ASP315	− 1.4 ± 2.1	0.4 ± 1.4	− 6.6 ± 8.4	5.0 ± 5.9	− 0.2 ± 0.1
PHE316	− 2.5 ± 0.6	− 2.5 ± 0.4	− 0.5 ± 0.4	0.9 ± 0.3	− 0.3 ± 0.1

**Fig. 8:**
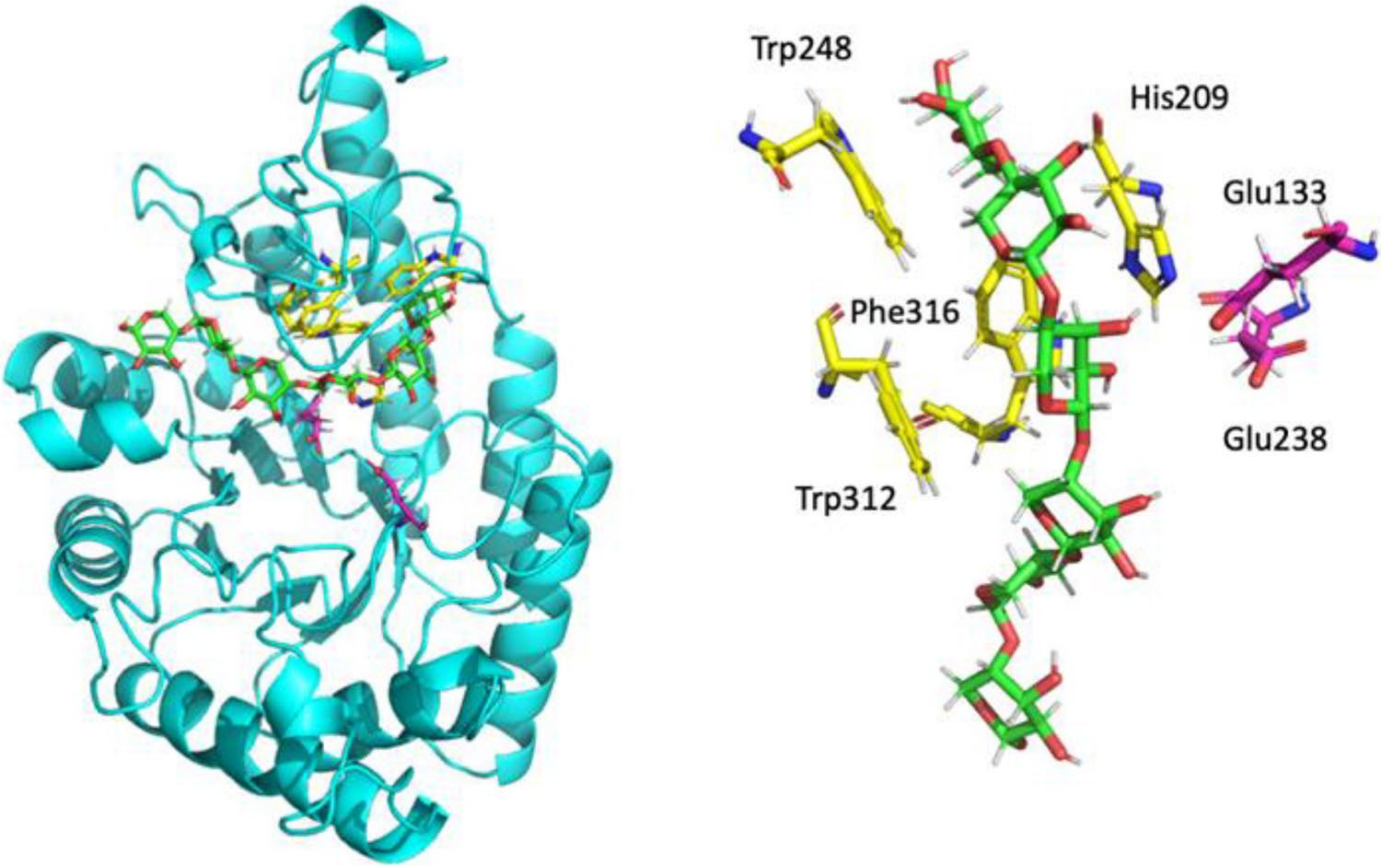
Representative structure from the MD simulations of the enzyme-xylohexaose complex. The relative position of the amino acid residues contributing most to the substrate binding free energy and the catalytic amino acid residues is also shown. The residues and substrate are shown as sticks with red indicating oxygen atom, blue corresponding to nitrogen atom, and green (substrate), pink (glutamate residues), or yellow (additional residues identified by MM-GBSA) for the carbon atoms

Figure 8 illustrates the interaction of the side chains of these amino acid residues with the substrates. Trp312 and Trp248 have a very high van der Waals contribution to substrate binding (− 4.4 and − 2.9 kcal/mol, respectively), corresponding to an almost planar positioning in relation to the xylose moieties of the substrate.

## Discussion

Hemicellulose is the most abundant component of the lignocellulosic residues after cellulose. The monomers that constitute the chains of this polysaccharide are bonded through glycosidic linkages. The demand for efficient glycosyl hydrolases with outstanding catalytic activity, namely hemicellulases, is crucial to facilitate the bioconversion of hemicellulose into several value-added products (Periyasamy et al. 2022; Nargotra et al. 2023). Xylanases with interesting catalytic features have been searched from extreme environments using metagenomic approaches (Sousa et al. 2022).

In this study, a xylanase from a compost metagenomic library was identified through a functional screening. A total of 12 out of the 563 screened clones showed a positive response for xylanase activity. Compared to other studies (Kwon et al. 2010; Colombo et al. 2016; Ellilä et al. 2019) evaluating the potential of compost metagenomes as a valuable source of xylanases, the proportion of positive clones in relation to the screened clones was higher in our work (2.1%). In fact, only 0.04% (Kwon et al. 2010), 0.13% (Colombo et al. 2016), and up to 0.05% (Ellilä et al. 2019) of positive clones were recorded in those studies. The great potential of our compost sample to find xylanases may be related not only to its composition but also to the origin of the selected biowastes (from domestic to public places). Since agro-industrial residues are already known to be an important source of xylan, the diversity—in terms of source and type—of the food, green, and forestry biomasses used in our compost sample may have increased the xylan levels and consequently contributed to induce the xylanase production (Amorim et al. 2019; Vitória et al. 2021). Regarding the results obtained in the functional screening at different temperatures (Fig. 1), a lack of response from some of the 12 clones previously identified as showing xylanase activity in the first screening may be due to the shorter incubation period defined for this assay. This shortening of the disclosure time from 1 week to 4 days aimed to select the clone(s) with the most quick and clear responses under different controlled temperatures. Furthermore, the absence of xylanase activity at 60 °C and the detection of the highest enzymatic activity at 37 °C may be related to the use of *E. coli* as host. Effectively, certain thermophilic enzymes may not be functional in this type of mesophilic host. The use of alternative host systems, such as *Thermus thermophilus*, can circumvent this problem (Angelov et al. 2009; Mirete et al. 2016; Wang et al. 2019) and should be considered in future metagenomic studies using composting samples. Nevertheless, two clones with xylanase activity (LP4 and LP11) were identified after 1 day of incubation over a slightly wide range of temperatures (between 25 and 45 °C), which makes them biotechnologically interesting. Based on the fast and clear response in the range of tested temperatures, LP4 clone was selected as the most promising one. In addition, this clone proved to be a xylanase producer under submerged fermentation. A xylanase activity of 5.62 ± 0.45 U/ml of crude intracellular enzyme was obtained, which is one of the highest values when compared to the study conducted by Kwon and co-workers using crude intracellular extracts of five xylanase positive clones from a compost metagenomic library and oat spelt xylan as substrate (0.2 to 13.1 U/ml) (Kwon et al. 2010). These promising results led to the analysis of the LP4 sequence, which confirmed that this protein is a GH10 endo-1,4-β-xylanase similar to that previously isolated from a compost metagenomic library prepared using pig manure and mushroom cultural wastes as raw materials (Jeong et al. 2012). Among the 50 β-xylanases classified as the most similar to the endo-1,4-β-xylanase identified in this study, and belonging exclusively to the GH10 family (Fig. 3), only a few of them were identified through metagenomic/metatranscriptomic studies, namely A0A7U1BNE3 1,4-β-xylanase, A0A7X8UBK4 β-xylanase, A0A2E8HSY2 β-xylanase, A0A2D8WRR4 β-xylanase, A0A2E4LF11 β-xylanase, A0A2V7SVW0 β-xylanase, Q5I2C6 xylanase, G0WRA6 β-xylanase, S5TC95 β-xylanase, and A0A2I6DH36 β-xylanase (Hu et al. 2008; Jeong et al. 2012; Alvarez et al. 2013; Diamond et al. 2018; Evangelista et al. 2018; Tully et al. 2018; Campanaro et al. 2020; Wang et al. 2021). From this pool, six were also collected from lignocellulose-rich sources, namely soil, composting, and pulp and paper manufacture wastewater, justifying their higher similarity to Xyl4 herein identified. In addition, 50% of the GH10 β-xylanases discovered from meta-omics approaches are derived from uncultured microorganisms. Nevertheless, most of the closest homologues, including the one with the greatest similarity, are β-xylanases from *Cellvibrio* species. In fact, bacteria from genus *Cellvibrio* are recognized to be aerobic and have been reported to play an important role in the degradation of different polysaccharides, among them xylan (Wu and He 2015; Blake et al. 2018). This is in accordance with the aerobic composting ecosystem from which our compost sample was collected.

As expected, the subcloning allowed to increase the production of the recombinant xylanase, Xyl4, since Xyl4 enzyme activity, without any purification step, increased about 2-fold when compared to the LP4 clone. The temperature (50 °C) and pH (approximately neutral) defined in this study to assess the catalytic activity of Xyl4 are in the same range as those revealed in other metagenome-derived GH10 xylanases, including the G0WRA6 β-xylanase that is optimally active at pH 7.0 and 40–50 °C. Other examples include rumen/gut microbial metagenomes (such as cattle and termite microbiota) with GH10 xylanases presenting optimal temperatures and pH in the ranges of 35–50 °C and 5.0–9.0, respectively (Cheng et al. 2012; Gong et al. 2013; Wang et al. 2015; Kim et al. 2018; Romero Victorica et al. 2020; Wu et al. 2021; Mon et al. 2022). More importantly, optimal temperatures of 40–80 °C and optimal pH between 5.5 and 9.0 of GH10 xylanases have been found for enzymes obtained from compost/soil metagenomes (Hu et al. 2008; Mo et al. 2010; Jeong et al. 2012; Weerachavangkul et al. 2012; Alvarez et al. 2013; Sun et al. 2015; Evangelista et al. 2018; Ellilä et al. 2019). In addition, these parameters, especially temperature, are consistent with the sampling point, since the samples were collected during the thermophilic phase (temperature > 45 °C) of the composting process, a high-temperature environment.

The IPTG, whose action is based on the *lac* operon, is one of the most popular expression systems in *E. coli* due to its extremely high expression levels. However, the use of higher concentrations of this inducer may affect the expression level and catalytic activity of enzymes (Zafar et al. 2016; Zhang et al. 2021). In this work, an increase in the IPTG concentration did not have an improved effect on enzyme expression, suggesting that 0.1 mM IPTG is a suitable concentration to induce expression. A possible explanation for this fact may include toxicity, saturation of the *lac* operon, or cell stress response promoted by high concentrations of IPTG, as previously reported (Dvorak et al. 2015; Gomes et al. 2020; Zhang et al. 2022).

Computational methods were applied to predict the Xyl4 structure and the interaction with the substrate, xylohexaose. The xylanases of GH10 family hydrolyze glycosidic bonds by acid-base catalysis through a double displacement mechanism involving two glutamate residues (Mendonça et al. 2023), positioned at the bottom of the active site cleft. This is consistent with the position observed for Glu133 and Glu238 in the arranged model. In addition, xylanases of this family typically have a substrate binding cleft extending along the length of the protein that can accommodate between four and seven xylose residues (Pell et al. 2004). This characteristic cleft was here identified and is comprised by residues that can form multiple hydrophobic and aromatic stacking interactions, as well as hydrogen bonds with xylosyl moieties in putative xylose substrates. The docking studies used the long cleft previously described, as a reference point for the exploration of the ligand interaction search space. All the three independent scoring functions predicted strong binding of xylohexaose to this cleft, with Glu133 and Glu238 interacting between moieties 3 and 4, at a distance that is consistent with catalysis and with a role of this enzyme as a xylanase. As the three scoring functions are based on different physical principles and approximations, the different scoring values cannot be compared directly with each other but must be compared against other molecules or targets evaluated with the same scoring function. Values in the range of 80–90 with ChemPLP, 68–72 with ASP, and 60–65 with Chemscore are indicative of strong association (Lapaillerie et al. 2022; Magalhães et al. 2022; Vieira et al. 2022; Fernandes et al. 2023). Most of the contribution of the most important residues identified by MM-GBSA to the binding free energy values arises from the van der Waals and non-polar component to the solvation free energy, indicative of the importance of π–π stacking and hydrophobic interactions between the side chains of these amino acid residues and the xylose rings. While hydrophobic interactions dominate the stabilization of xylose, for catalysis, the introduction of changes at the active site that could lead to a further stabilization of the substrate in the proximity and orientation of Glu133 and Glu238 would be essential to improve the xylanase activity of this enzyme. This change could be done by the introduction of more volume around the active site, opposite to the glutamate position. An inspection of the active site suggests that the replacement of Phe316 by a the bulkier Trp could be a promising first step to achieve this goal.

In conclusion, this study confirms that extreme environments, particularly composting, are excellent sources to find robust xylanases. The functional screening of a large insert compost metagenomic library led to the discovery of a xylanase gene encoding a xylanase (Xyl4) belonging to the GH10 family. The significant number of positive xylanase clones identified allows to conclude that the composition and origin of our compost sample make it a source with high potential to find xylanases. The most promising positive clone for xylanase activity (LP4) was selected through an innovative two-step screening using a developed pipeline that involves MATLAB’s in-house functions. This selection considered the development of larger blue halos in a shorter period and over a slightly wide range of temperatures. The selected LP4 clone was evaluated as an effective xylanase producer under submerged fermentation conditions and a GH10 endo-1,4-β-xylanase, encoded by *xyl4*, was identified. After subcloning and expression, the recombinant Xyl4 showed higher levels of hydrolytic activity when compared to the LP4 clone at high temperature. Computational analyses were applied to give an insight into the structure of Xyl4 and to reveal critical information on its binding with the xylohexaose substrate, suggesting, for the first time, potential strategies to engineer the active site of Xyl4 and enhance its enzymatic activity. Xylanases are valuable biocatalysts with several industrial applications, including the valorization of lignocellulosic residues (Ajeje et al. 2021). Therefore, efforts to enhance their enzymatic activities will greatly contribute to implement biorefinery and circular economy practices and meet the green industrial policy. Improved xylanases will have a crucial role in the efficient conversion of hemicellulose-rich biomass into a spectrum of value-added products (e.g., biofuels or prebiotic sugars). Similarly, other industrial sectors such as the food and feed, pulp and paper, or textile can also benefit for their enhanced catalytic activity.

## Data Availability

The authors confirm that the datasets supporting the findings and conclusions of this study are available within the article. Additional data can be provided upon request.
